# The Effect of Parathion on Red Blood Cell Acetylcholinesterase in the Wistar Rat

**DOI:** 10.1155/2016/4576952

**Published:** 2016-06-23

**Authors:** Naofumi Bunya, Keigo Sawamoto, Hanif Benoit, Steven B. Bird

**Affiliations:** ^1^Sapporo Medical University, Sapporo 060-8556, Japan; ^2^Department of Emergency Medicine, University of Massachusetts Medical School, Worcester, MA 01655, USA

## Abstract

Organophosphorus (OP) pesticide poisoning is a significant problem worldwide. Research into new antidotes for these acetylcholinesterase inhibitors, and even optimal doses for current therapies, is hindered by a lack of standardized animal models. In this study, we sought to characterize the effects of the OP pesticide parathion on acetylcholinesterase in a Wistar rat model that included comprehensive medical care.* Methods*. Male Wistar rats were intubated and mechanically ventilated and then poisoned with between 20 mg/kg and 60 mg/kg of intravenous parathion. Upon developing signs of poisoning, the rats were treated with standard critical care, including atropine, pralidoxime chloride, and midazolam, for up to 48 hours. Acetylcholinesterase activity was determined serially for up to 8 days after poisoning.* Results*. At all doses of parathion, maximal depression of acetylcholinesterase occurred at 3 hours after poisoning. Acetylcholinesterase recovered to nearly 50% of baseline activity by day 4 in the 20 mg/kg cohort and by day 5 in the 40 and 60 mg/kg cohorts. At day 8, most rats' acetylcholinesterase had recovered to roughly 70% of baseline. These data should be useful in developing rodent models of acute OP pesticide poisoning.

## 1. Introduction

Organophosphorus (OPs) pesticides were developed in the 1930s. By the year 2000, approximately 1 billion pounds of pesticides was used annually in the United States, with worldwide use estimated to by more than 5.5 billion pounds [1]. OP pesticides irreversibly bind to and inhibit acetylcholinesterase (AChE). AChE is an enzyme that catalyzes the breakdown of acetylcholine (Ach) to choline and acetic acid. During neurotransmission, ACh is released from the presynaptic neuron into the synaptic cleft and binds to ACh receptors on the postsynaptic membrane, thereby propagating the signal from the nerve. AChE (which is located at the neuromuscular junction, central and peripheral nervous tissues, and red blood cells) terminates the nerve signal transmission by hydrolyzing ACh [2]. Therefore, AChE inhibition by OP pesticides leads to a widely dispersed accumulation of ACh. This increase in ACh manifests acutely as the cholinergic syndrome: bradycardia; central respiratory failure; and bronchorrhea and bronchospasm [3–5]. To examine the time course of AChE after OP poisoning, we needed to overcome this acute cholinergic syndrome. For this reason, the rats needed to be supported with a mechanical ventilator and comprehensive medical therapy.

In part because the OP pesticides are used to such a large degree in developing countries, there is significant mortality due to OP pesticide poisoning. The World Health Organization has estimated that more than 3 million people suffer from pesticide poisoning each year, with a case fatality rate of up to 10% [6]. These numbers do not take into account estimates that self-poisoning with pesticides accounts for more than 250,000 deaths per year worldwide, accounting for about one-third of the world's suicides [7]. Aside from acute mortality, it is now known that significant morbidity such as muscle weakness and neurocognitive dysfunction occurs after OP pesticide poisoning [8–10].

Part of the difficulty in studying both the acute and chronic effects of OP pesticides is the lack of animal models of OP poisoning and infrequent reporting of AChE activity and variable dosing and formulation of various pesticides [11, 12]. The aim of this investigation was to determine the effect of AChE of various doses of the highly toxic OP pesticide parathion, in order to determine the LD50 of parathion in a validated OP pesticide poisoning rodent model utilizing comprehensive intensive care therapy.

## 2. Material and Methods

Male Wistar rats weighing 200–350 gm (Charles River Laboratories, Wilmington, MA) were housed in pairs, maintained on 12 : 12 light : dark cycle, and provided food and water* ad libitum*. Parathion-ethyl (Sigma-Aldrich, St. Louis, MO) was administered at three different doses: 20 mg/kg, 40 mg/kg, and 60 mg/kg. The scientific literature is not consistent when determining the LD50 of toxic OPs in animal models. For instance, various strains of rodents have been used; the OP pesticide has been administered by various routes; and most importantly, comprehensive medical therapy (including mechanical ventilation) has rarely been utilized. It is worth noting that the LD50 for parathion has been reported to be as low as 2 mg/kg in the rat [13, 14].

Rats were briefly anesthetized for 1-2 minutes with isoflurane. Once anesthetized, endotracheal intubation was performed using a 14- or 16-gauge Teflon catheter. An internal jugular catheter of PE-50 tubing was placed and tunneled subcutaneously to between the scapulae. A 24-gauge catheter was also placed in a tail vein. General anesthesia was maintained with 2–4% isoflurane and oxygenation saturation, heart rate, noninvasive blood pressure, and temperature (with feedback to a heating pad) were monitored. Mechanical ventilation was performed using various rodent ventilators (Harvard Apparatus, Braintree, MA) with intermittent capnography. Once ventilation was stabilized, parathion was administered via the jugular vein catheter at a dose of 20 mg/kg (20 rats), 40 mg/kg (35 rats), or 60 mg/kg (37 rats).

When bradycardia developed (defined as a decrease in heart rate of 50% from baseline) atropine was given as a bolus dose of either 0.1 or 0.6 mg/kg followed by a continuous infusion of either 40 or 250 mcg/kg/h via the jugular vein. In order to mimic a human poisoning scenario, where comprehensive medical therapy is used, 0.125 mg/kg of midazolam (Patterson Veterinary Supply, Devens, MA) was given subcutaneously and every 4 hours. Pralidoxime chloride (2-PAM, Sigma-Aldrich, St. Louis, MO) was administered at a dose of either 15 mg/kg or 90 mg/kg every 6 hours via tail vein. The higher doses of atropine and pralidoxime were based upon recent guidance from the NIH to use species-specific modifications of human medication doses [15]. The pralidoxime dosing interval was chosen based upon typical dosing regimens used in the United States. Because pralidoxime is standard therapy in the United States, the Institutional Animal Care and Use Committee of the University of Massachusetts Medical School only approved this study with the utilization of pralidoxime. Maintenance intravenous fluid of normal saline (1.5 mL/kg/h continuously) via tail vein route was given and the body temperature of 37-38 degrees Celsius was maintained throughout.

Twenty-four hours after poisoning the ventilator was stopped and the rats were extubated. In order to counteract the continued cholinergic signs atropine was infused continuously via the tunneled jugular catheter for another 24–48 hours in an infusion chamber (Instech, Plymouth Meeting, PA). At day 3 or 4 after poisoning (dependent upon a rat's condition), the animal was moved to normal housing container. Not all rats survived the poisoning model, but survivors were euthanized at day 8 after poisoning.

Animal ChE Test System Model 610 (EQM Research, Cincinnati, OH) was used to measure AChE from blood expressed from the tail vein. The Animal ChE Test System is a validated desktop AChE measurement system that allows AChE determination from just 10 *μ*L of blood. The AChE reagent is >95% specific due to the addition of a specific inhibitor of plasma butyrylcholinesterase (PChE), As1397 (10-(a-diethylaminopropionyl)-phenothiazine). In order to minimize any interactions between parathion, pralidoxime, and AChE, all blood samples were analyzed immediately upon obtaining the blood.

All statistical analyses were performed with GraphPad Prism software version 4 (GraphPad Software, San Diego, CA).

## 3. Results

**Figure 1:**
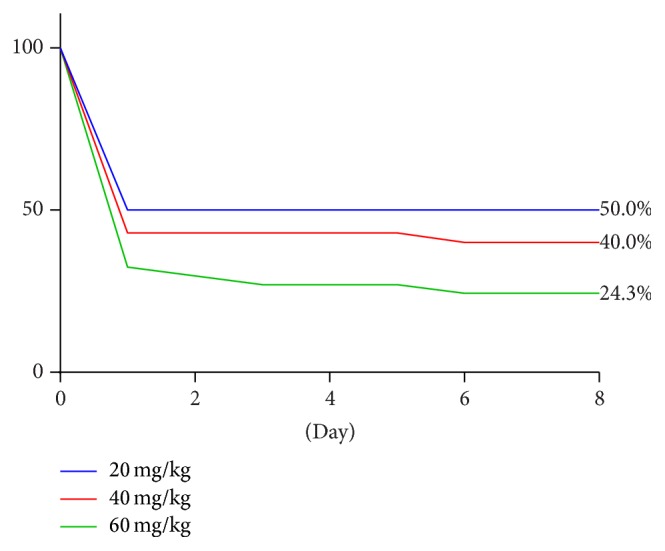
Kaplan-Meier survival curves for various doses of intravenous parathion.

Of the 20 rats given 20 mg/kg parathion, 10 survived to day 8. At 40 mg/kg, 14 rats out of 35 survived, and at 60 mg/kg parathion 9 rats out of 37 survived.  Figure 1 is a Kaplan-Meier survival curve after poisoning with various doses of parathion.

Of the rats that did not survive parathion doses from 20 to 60 mg/kg, nearly all (93%) were dead within 24 hours of poisoning or proximate to the time of extubation (within 1 to 2 hours). One rat was dead at day 6 in the 40 mg/kg parathion group. Two rats were dead at day 3 and one rat was dead at day 6 in the 60 mg/kg parathion group. Because nonsurviving rats typically died within 24 hours of poisoning, no further AChE data could be obtained. However, AChE activity was neither lower nor more rapidly inhibited in the nonsurvivors. Furthermore, these animals generally looked very weak but did not appear to have more cholinergic signs than surviving rats.

**Figure 2:**
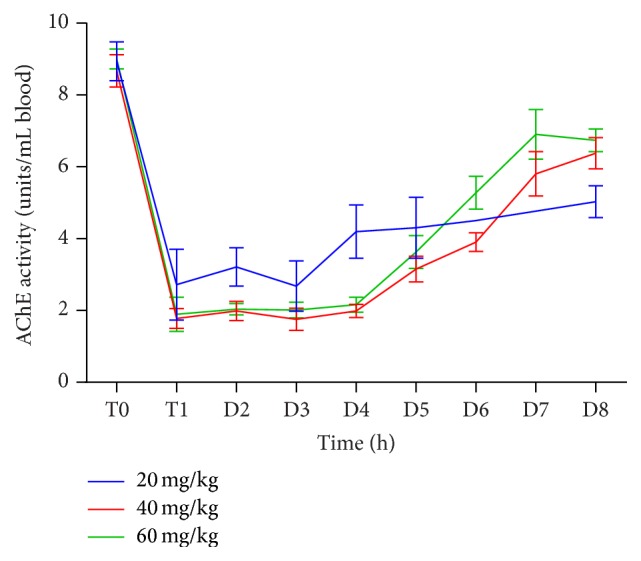
AChE activity after poisoning with various doses of IV parathion.

AChE activity over a period of 8 days after poisoning for all parathion doses in surviving rats is shown in Figure 2. AChE recovered to nearly 50% of baseline activity by day 4 in the 20 mg/kg cohort and by day 5 in the 40 and 60 mg/kg cohorts. At day 8, most rats' AChE recovered to roughly 70% of baseline.

**Figure 3:**
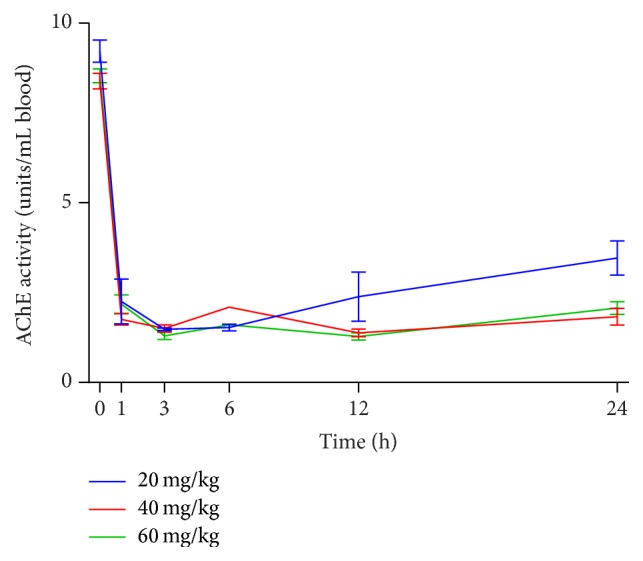
AChE activity over the first 24 hours after poisoning with various doses of IV parathion.

Figure 3 shows AChE activity over the first 24 hours after parathion poisoning in all rats and for parathion doses from 20 mg/kg to 60 mg/kg. All rats were grouped together regardless of the pralidoxime dose received (either 15 or 90 mg/kg), as there was no statistically significant difference observed in AChE activity based upon dose of pralidoxime (data not shown).

For all doses studied, AChE decreases to the lowest point at 3 hours after parathion poisoning. Rats in the 20 mg/kg parathion group began to recover AChE at 12 hours, whereas rats in the 40 and 60 mg/kg groups recovered AChE to a statistically significant degree at 24 hours after poisoning. However, the area under the curve (AUC) of AChE activity over 24 hours was not statistically different among the 20, 40, or 60 mg/kg parathion cohorts.

## 4. Discussion

Data from these studies demonstrate that in this rat model AChE activity is inhibited very quickly and in a dose dependent manner, with a nadir of AChE occurring at approximately 3 hours after poisoning regardless of the dose of parathion administered. In animals that were alive 8 days after poisoning, AChE had only returned to approximately 70% of the baseline activity, regardless of the parathion dose used. Furthermore, there was no difference observed in AChE activity in animals that received either 15 mg/kg or 90 mg/kg 2-PAM.

This seeming indifference to the dose of 2-PAM is not unexpected. The true value of 2-PAM in the treatment of acute OP poisoning is the subject of significant debate. Mechanistically, pralidoxime dose reactivates OP-inhibited AChE and improves neuromuscular transmission in* ex vivo* models [16, 17]. However, while pralidoxime has for years been considered standard therapy for acute OP poisoning and has been shown to be beneficial in some animal models [18] and human studies of OP poisoning [19], these results are not universal. For instance, several clinical trials of pralidoxime have failed to show benefit (or trended towards harm) in patients acutely poisoned with OP pesticides [20–23]. While the possible reasons for why pralidoxime may or may not be beneficial in humans poisoned by OP pesticides are beyond the scope of this paper, there are certainly many factors simultaneously at play, including the time from poisoning to treatment; the type of OP pesticide (e.g., dimethyl versus diethyl OP); the lipophilicity of the OP pesticide; and the dose of pralidoxime. Furthermore, there may be a threshold dose and effect for pralidoxime, and using doses above this threshold does not improve AChE reactivation.

Few animal models of acute OP pesticide poisoning exist. This has been particularly true with rodents, and no previous studies have looked at comprehensive medical therapy to include mechanical ventilation. This study is important as it allows us to obtain biochemical and survival data in rodents treated identically to poisoned humans. Developing these important animal models has been hindered by the variability of the models and the lack of studying or reporting meaningful data in these models. For example, depending on the age, gender, and rodent species used, the oral LD50 of parathion has been reported to be between 2 mg/kg and 30 mg/kg [14]. Such extreme variability makes it virtually impossible to draw any meaningful conclusions or to develop a realistic animal model. We chose our doses of parathion to span these reported LD50s. Furthermore, LD50s have been reported in animals exposed to parathion without any medical therapy. But in order to test new therapies, it is important to know what the LD50 is in animals given comprehensive medical therapy. Lastly, nearly all animal studies of OP pesticide poisoning fail to report AChE activity. Thus, until now the true LD50 of parathion* during medical treatment* has been unknown.

However, rodent and other models serve a critically important purpose with acetylcholinesterase inhibitor research. The US Food and Drug Administration employs what has been termed the “animal rule” in order to approve therapies aimed at reducing or preventing serious or life-threatening conditions caused by exposure to a “permanently disabling toxic agent,” such as OP pesticides, in which human efficacy trials are not possible or ethical [12]. Based on this animal rule, the FDA may rely exclusively on animal efficacy studies to provide evidence of therapeutic [24]. While the animal rule is relatively new and underutilized for drug deployment, it was invoked by the FDA in 2003 for the approval of pyridostigmine bromide as a pretreatment for nerve agent exposure in military personnel [25].

As expected with any toxin, there was a dose dependent decrease in survival in this model, even with critical care support. At a parathion dose of 20 mg/kg, exactly one-half of animals survived to 24 hours and to 8 days after poisoning. At a dose of 60 mg/kg parathion 24.3% of animals survived: thus the LD75 for IV parathion is approximately 60 mg/kg. This is important, as is toxicology survival research in animal models: a toxin dose of greater than a single LD50 is needed in order to detect clinically and statistically meaningful results with a minimal number of animals. It appears that 3x LD50 of parathion (i.e., 60 mg/kg intravenously) may be a reasonable dose for further survival studies.

## 5. Conclusion

In the Wistar rat, parathion inhibits AChE in a dose dependent manner, with the AChE nadir occurring at approximately 3 hours after IV parathion administration regardless of the parathion dose used. Using comprehensive critical care therapy, including mechanical ventilation, atropine, pralidoxime, and midazolam, the IV LD50 for parathion is approximately 20 mg/kg. These data will be useful for investigators developing models of acute OP poisoning and studying treatment algorithms for acute poisoning.
